# The Coming of the Mob

**Published:** 1921-03-19

**Authors:** 


### THE EDITOR'S LETTER-BOX.

### THE COMING OF THE MOB.

*To the Editor of* The Hospital.

Sir,—Nurses have more than once been compared to a flock of sheep, apparently because a characteristic trait of that gentle species is that they may be driven or led without resistance into pleasant or unpleasant places. I do not think the description is quite accurate. What more probably happens is that the average nurse remains passive, thinking her own thoughts, usually without expressing them, and leaving her fate in the hands of those who have constituted themselves her leaders.

It is a somewhat mournful spectacle to contemplate, and for the reason that the leaders (or drivers) include people whose vision is defective, and their land of promise sometimes proves when too late to be a dismal swamp. I am thinking of this post-war godsend, yclept Registration, part of the reconstruction, redemption, and emancipation scheme to reward nurses for their heroism and sacrifice. You remember the turmoil and the clamour. Anybody who ventured to demur was denounced as a traitor and a self-seeker. So loud and persistent became the noise that even people who saw the utter folly of the whole business began to wonder if, after all, their judgment was unsound.

The murder is now out, and in a nut-shell it amounts to this, that Registration cannot by any possibility benefit trained nurses now living. So far from this being the case it will of necessity do them very serious injury. The flood-gates are about to open, and they will remain open for two years, during which period a great multitude of persons engaged in nursing, but classed as untrained, will be swept in. Hence anybody who can get certified will be entitled to wear the title Registered Nurse, and, presumably, will become eligible for appointments in the Army, Navy, or Municipality, hitherto open only to the trained nurse. I do not need to be reminded that the admission of the half-qualified is incidental to all Registration measures, nor does it afford any consolation to know that when we are dead these Acts will begin to bear good fruit. A mob of untrained nurses are about to be decorated with the badge of qualification, and neither the public nor the profession will be able to discriminate after a few months have passed. It is very desirable that the trained nurse should appreciate what has occurred.

I am in hope that the College may be able to provide a remedy of some sort. Even if it is not wholly effectual, something is better than nothing. The College is an institution whose title suggests culture and the recognition of educational attainments. It is certainly due to those women who have served a strenuous apprenticeship and passed their various examinations that they should be segregated from the crowd who, until to-day, have been by common consent catalogued as untrained. It may seem an ironical suggestion that something should be done to redress the wrongs of Registration even before it becomes operative. But the facts speak very eloquently, and I do not know how anybody who is concerned with the interests of the trained nurse can regard them with equanimity.

Moreover, if one may venture to speculate, it seems highly probable that for new entrants the future standard of qualification will be low. The "one portal," which was the main plank of the Registrationists' platform, makes this unavoidable. These war-cries sounded very plausible and democratic when Registration was only a day-dream. Now that it comes into the region of reality they stand for retrogression and demoralisation, and must continue so to do until registered nurses are classified and properly graded. One of the most serious defects of trade unionism amongst craftsmen is the rule that all workers must be paid a fixed wage. Employers are debarred from rewarding skill and industry. The effect is blighting, and one consequence is the inflated price of commodities. Every member of the community is the victim of this tyranny.

Unless trained nurses lift up their voices and insist on a remedy being provided they will find themselves very frequently outclassed and outmanœuvred by substantially inferior competitors.—Yours faithfully,

